# Determination of (-)-epigallocatechin-3-gallate octaacetate and its metabolites in plasma of rats for pharmacokinetic study by ultra-performance-liquid-chromatography coupled to quadrupole-time-of-flight-mass-spectrometry

**DOI:** 10.3389/fphar.2022.1025053

**Published:** 2022-10-11

**Authors:** Kai On Chu, Gene Chi Wai Man, Sze Wan Hung, Tak Hang Chan, Wai Yip Thomas Lee, Kwok Ping Chan, Chi Pui Pang, Chi Chiu Wang

**Affiliations:** ^1^ Department of Ophthalmology and Visual Sciences, The Chinese University of Hong Kong, Shatin, China; ^2^ Department of Obstetrics and Gynaecology, The Chinese University of Hong Kong, Shatin, China; ^3^ Department of Applied Biology and Chemical Technology, The Hong Kong Polytechnic University, Hong Kong, China; ^4^ Department of Chemistry, McGill University, Montreal, QC, Canada; ^5^ Aptorum Group Limited, Hong Kong, China; ^6^ Joint Shantou International Eye Center of Shantou University and the Chinese University of Hong Kong, Shantou, China; ^7^ Li Ka Shing Institute of Health Sciences; and School of Biomedical Sciences, The Chinese University of Hong Kong, Shatin, China

**Keywords:** (-)-Epigallocatechin-3-gallate-octaacetate, LC-MS, determination, validation, pharmacokinetics

## Abstract

(-)-Epigallocatechin-gallate octaacetate (pro-EGCG), a prodrug of epigallocatechin-gallate (EGCG), has been used for pre-clinical study for the treatment of endometriosis. A validated analytical method has been developed for the determination of plasma pro-EGCG and its metabolites after oral administration using ultra-performance-liquid-chromatography coupled to quadrupole-time-of-flight-mass-spectrometry (UPLC-Qtof-MS). This method is more robust, rapid, sensitive, simpler, and able to detect pro-EGCG metabolites compared to our previous method. Pro-EGCG in the plasma was stabilized from rapid degradation by formic acid, extracted by isopropanol/methyl-tert-butyl ether mixture, separated by UPLC core column, and quantified by an exact mass method with Qtof-MS. The lower limit of quantification (LLOQ), intra-day and inter-day precision, and accuracy for the range of 0.01–2.5 μg/mL were within acceptable limits. The sensitivity was improved by 25 folds using pro-EGCG ammonium adduct [M + NH4]^+^. This is the first report on the pharmacokinetics of oral administration with maximum-concentration (Cmax) was 0.067 ± 0.04 μg/mL, time-of-maximum-concentration (Tmax) was 1.33 h, area-under-curve (AUC) was 0.20 ± 0.05 h × µg/mL, and elimination-rate was 0.20 ± 0.11 hr^−1^. The pharmacokinetic profiles of pro-EGCG metabolites, (-)-epigallocatechin-gallate (EGCG) diacetates and EGCG triacetates, were also presented. This method is robust, rapid, and sensitive for the pharmacokinetic study of pro-EGCG and metabolites.

## Graphical Abstract

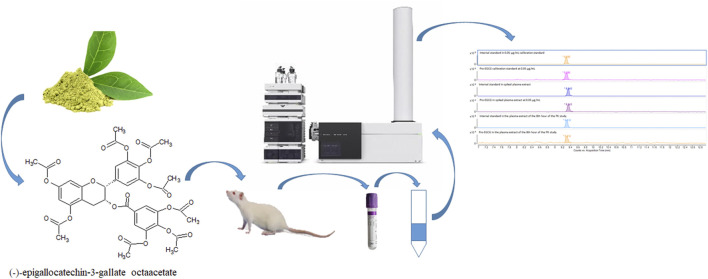

## 1 Introduction

Tea is the second most popular beverage worldwide after water (Heiss and Heiss, 2007). It has been extensively studied for therapeutic uses because of its anti-mutagenic (Bunkova et al., 2005), anti-inflammatory (Thangapazham et al., 2007), anti-oxidative, anti-microbial (Suzuki et al., 2012), anti-carcinogenic (Ferrazzano et al., 2011), and anti-angiogenesis properties (Wang et al., 2013) mainly attributed to its catechin constituents. Recently, green tea extract has been used to inhibit the main protease of SAR-CoV-2 to combat COVID-19 (Ghosh et al., 2020; Montone et al., 2021). Epigallocatechin gallate (EGCG) is the most prominent catechin in tea as it is the highest in concentration and most potent in biological activities among the natural green tea catechins. In human cells, anti-apoptosis by EGCG has been reported (Rajabi et al., 2021). Meanwhile, anti-neoplastic properties for prostate in clinical studies have been inconsistent (Choan et al., 2005). The bioavailability of EGCG is not adequate for the biological effects *in vivo* possibly due to its chemical instability (Chen et al., 2001) and extensive metabolism (Lambert et al., 2003).

**FIGURE 1:**
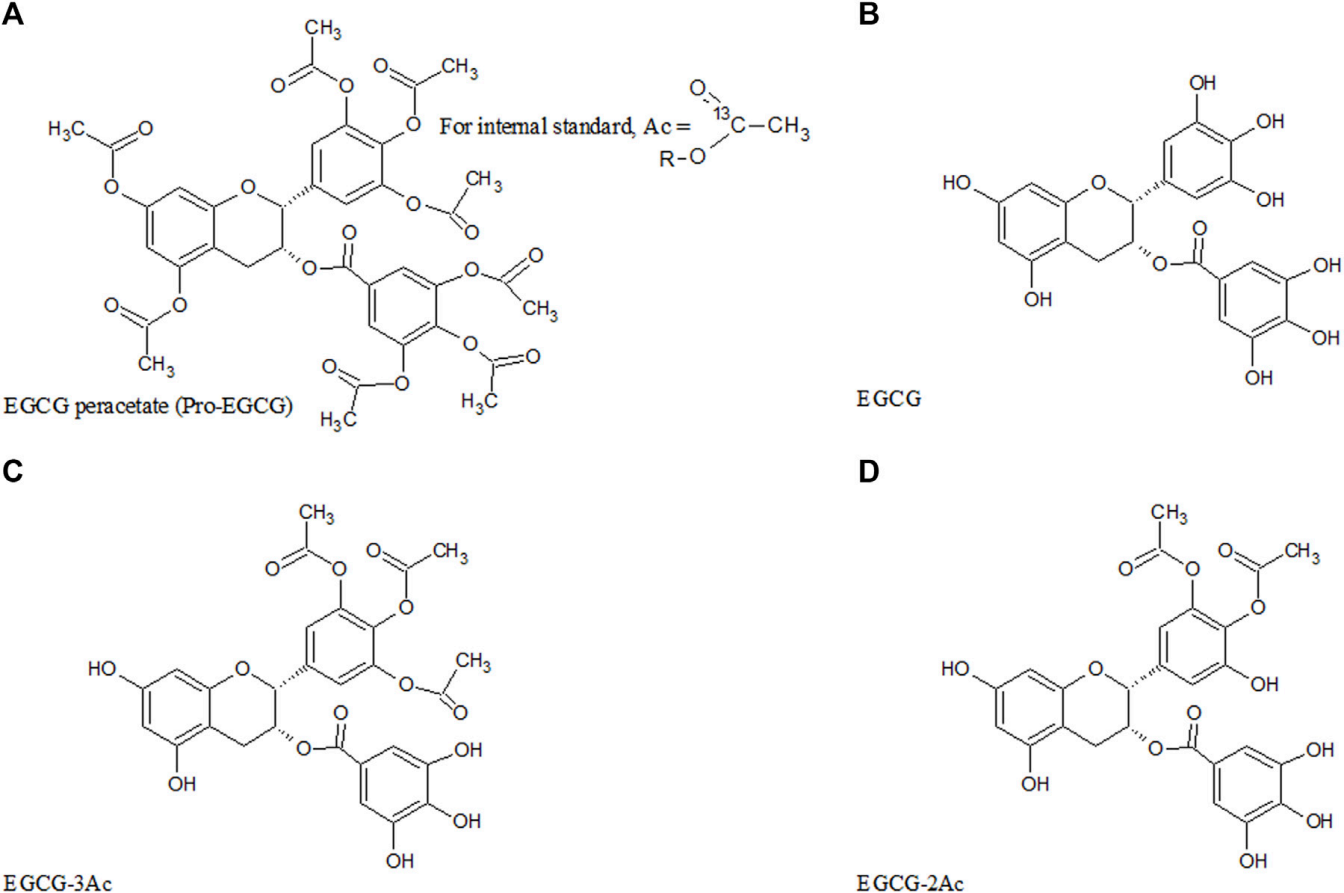
Chemical structure of pro-EGCG and metabolites. Diagrams show the chemical structure of **(A)** pro-EGCG, **(B)** EGCG, and the proposed structure of isomer of **(C)** EGCG-3Ac, and **(D)** EGCG-2Ac.

A pro-drug of EGCG, EGCG octaacetate (pro-EGCG), obtained by acetylation of EGCG (Figure 1), has been shown to have higher resistance to acid/base hydrolysis and so should have higher bioavailability than EGCG. It has been tested as a potential pharmaceutical drug for the treatment of various diseases in pre-clinical trial studies including endometriosis (Hung et al., 2021; USSEC, 2021). It possesses efficacious proteasome inhibitory and anticancer activities that have also been shown in human breast cancer cells and mouse tumor tissues (Landis-Piwowar et al., 2007). In animal models, pro-EGCG has more than two-fold potency compared to the equivalent amount of EGCG in inhibiting the proteasome activity of breast cancer cells, MDA-MB231 (Landis-Piwowar et al., 2007). In nude mice with induced prostate cancer, the tumor volume was 40.1% smaller after pro-EGCG treatment than EGCG treatment (Lee et al., 2008). In mice with induced skin tumors, there were three folds lower tumor incidences and three folds lower the average number of tumors per mouse (Chiou et al., 2013). The half-maximal inhibitory concentration (IC50) of clonogenicity of the human lung cancer H460 cells by pro-EGCG treatment was slightly lower than by EGCG treatment (Heyza et al., 2018). In a mouse model with induced endometriotic lesions, the plasma vascular endothelial growth factor (VEGF) concentration treated by pro-EGCG was two-thirds of the concentration treated by EGCG (Wang et al., 2013). Pro-EGCG not only inhibited VEGF secreted by endometrial cancer cells but also reduced the secretion by tumor-associated macrophages (Wang et al., 2018).

Pro-EGCG exhibited different stabilities *in vitro* and *in vivo* studies. Pro-EGCG is stable outside the cells but deacetylated to EGCG inside the cells (Lam et al., 2004). Hence, pro-EGCG could be determined directly by HPLC–UV from the culture medium (Lam et al., 2004). On the other hand, the plasma levels of pro-EGCG in *ex vivo* plasma and experimental animal models were very low due to enzymatic degradation (Lambert et al., 2006; Chu et al., 2014). Besides, limited blood volume from living rats, high protein binding, and complicated matrix render determining the plasma level of pro-EGCG from *in vivo* study to be difficult. Intragastric administration of pro-EGCG to CF-1 mice had resulted in higher bioavailability of EGCG compared with EGCG administered at equimolar doses (Lambert et al., 2006). However, the oral bioavailability of pro-EGCG is expectedly low owing to its extensive acetylated structure which is highly susceptible to hydrolysis by esterases in the blood, and gastric and intestinal fluid. Furthermore, the detection in plasma and other biological fluids is technically difficult because of its high hydrophobicity (Chu et al., 2014). Nevertheless, oral administration would be a preferable route because of convenience and non-invasiveness. Owing to insolubility in gastric fluid, pro-EGCG could be slowly released to maintain a sustainable profile. Because of enzymatic degradation and limited water solubility in the gastrointestinal system, we need to establish a systematic pharmacokinetic protocol to confirm the bioavailability and determine the optimal dosage. So far there is no published pharmacokinetic study of pro-EGCG for oral administration because it requires a rapid, high sensitivity, and specificity analytical method for the determination.

Previously we have developed a method to quantify pro-EGCG in mouse plasma following parenteral administration using thin-layer chromatography (TLC) purification, and micro-liquid chromatography - quadrupole time-of-flight (LC-QTof) mass spectrometry for determination (Chu et al., 2014). However, this method was not sufficiently sensitive for pharmacokinetic studies following oral administration. Also, it requires a labor-intensive TLC clean-up procedure, cannot retain metabolites of pro-EGCG, and cannot preserve pro-EGCG from rapid enzymatic degradation after plasma sampling. To improve the method, we added formic acid to the plasma to preserve pro-EGCG and used a high-efficiency UPLC core column to eliminate the TLC clean-up step and retain pro-EGCG metabolites. We applied UPLC-QTof-MS to replace micro-LC- QTof-MS in conventional method to improve the ruggedness, throughput, and separation efficiency. The ammonium adduct of pro-EGCG was selected to replace the hydrogen adduct to increase the sensitivity of the quantification.

## 2 Materials and methods

### 2.1 Chemicals and reagents

Acetonitrile and methanol were LC/MS-grade from J. T. Baker (Central Valley, PA). Formic acid was SuperPur grade from Merck (Darmstadt, Germany). Methyl-tert-butyl ether (MTBE) was HPLC plus grade from Sigma (St. Louis, MO). Iso-propanol (IPA) was HPLC grade from RCI Labscan (Rue Comot, France). Pro-EGCG was synthesized according to a reported procedure (Lam et al., 2004). The internal standard EGCG-octaacetate-13C8 was synthesized as published (Chu et al., 2014). The esterase inhibitors, 4-(2-aminoethyl)-benzenesulponyl fluoride HCL (pefabloc), bis-nitophenyl phosphate (BNPP), N-α-tosyl-L-lysine-chloromethylketone HCL (TLCK), dichlorvos, sodium fluoride, potassium oxalate (NaF-KO) were obtained from Sigma (St. Louis, MO). All other chemicals used were at their highest grade.

### 2.2 Animals

Fourteen to 16-weeks old female Sprague Dawley (SD) rats at reproductive age and of approximately 250 g were grouped in different cages in an air-conditioned room (22 ± 2°C) with a 12-h controlled lighting cycle. The SD rats were fed with standard chow and given free access to water. They were acclimatized for 1 week before the experiment. The study was approved by the Animal Experimentation Ethics Committee of the Chinese University of Hong Kong and was performed according to institutional guidelines. For validation studies, the rats were fasted overnight and then anesthetized by inhaling isoflurane (Baxter, IL, United States) for 15 min. Blank blood samples, approximately 10 mL, were withdrawn by cardiac puncture after experiments and before sacrifice.

### 2.3 Sample preparations

Venous blood drawn from the SD rats was stored in an EDTA tube in an -10°C ice bath for not more than 10 min and centrifuged at 1780 × g and -10°C for 10 min. Plasma was aliquoted in 50 μL, mixed with 6 µL formic acid, and either immediately processed or stored at -80°C.

The extraction procedure was modified from our previous study (Chu et al., 2014). A plasma aliquot was spiked with 2.5 µL of 100 μg/mL pro-EGCG internal standard and mixed with 300 µL of isopropanol and 3 µL of formic acid. After vortexing for 2 min and sonicating for 2 min, 1 mL of MTBE and 150 µL magnesium chloride 0.043% solution were added. The mixture was again vortexed for 2 min and sonicated for 2 min. After settling, the mixture was centrifuged at 2,624 × g and 4°C for 10 min. The supernatant was pipetted into a glass tube by a glass Pasteur pipette and avoided touching the protein layer. The remaining aqueous layer was re-extracted by isopropanol and MTBE as described above. The supernatant was mixed with the previous extract and purged to dryness by nitrogen. The dried residues were dissolved in 100 µL methanol, sonicated for 30 s, vortexed for 2 min, and centrifuged at 3,634 × g at 4°C for 15 min. The supernatant was transferred to an HPLC vial for LCMS analysis. Methanol can completely dissolve pro-EGCG and precipitate protein matrix from the residues.

### 2.4 LC-MS conditions

Plasma extract was analyzed by Agilent 6,540 Liquid chromatography-electrospray ionization quadrupole-TOF Mass Spectrometer. The extract, 8 μL, was separated by Waters Cortex UPLC C18 1.6 µm × 2.1 × 150 mm column (Waters, Milford, MA). Mobile phase A was 0.1 mM ammonia solution/acetonitrile/methanol/formic acid [80:15:5:0.2 (v/v/v/v)] and mobile phase B acetonitrile/methanol/0.1 mM ammonia solution/formic acid [92.5:5:2.5:0.2 (v/v/v/v)]. The column temperature was 40°C and the flow rate was 0.4 mL/min. The binary elusion program: 20% B, 0–2 min; 20%–60% B, 2–10 min; 60%–100% B, 10–11 min; 100% B, 11–18 min; 100%–20% B, 18–19 min; 20% B, 19–24 min. Positive electrospray ionization was used with capillary voltage (VCap) at 4500V, nozzle voltage at 1000 V, the gas temperature at 300°C, drying gas at 8 L/min, nebulizer pressure at 40 psi, sheath gas temperature at 300°C, and sheath gas flow at 11 L/min. The acquisition range was 100–1,000 m/z. The exact mass method was used for quantification at the range of 812.2043 ± 0.06 m/z for pro-EGCG ammonium adduct [M + NH4]^+^ and 803.2039 ± 0.06 m/z for internal standard pro-EGCG-1–13C8 hydrogen adduct [M + H]^+^. The mass was locked by continuous infusion of calibration standards with lock mass calibrated at 922.0 m/z.

For analysis of the metabolites, the predicted masses of pro-EGCG metabolites as calculated from de-acetylation, glucuronidation, sulphation, and methylation were scanned from the chromatogram of the first-hour plasma samples after intragastric administration of pro-EGCG. For a particular metabolite, the predicted target molecular mass of the metabolite was submitted for MS/MS analysis within ± 1 min around the retention time of the peak. The chromatography and mass spectrum conditions were the same as above. The collision energy was set for 10–20 mV and the daughter ion scan ranged from 100–1,000 Da. The molecular identity was elucidated by the accurate mass and MS/MS fragments because no commercial standard was available for comparison.

### 2.5 Validation

The method has been validated to analyze pro-EGCG in rat plasma. Since the concentration of pro-EGCG in plasma is relatively low, for the fit-for-purpose principle, we used a narrower linear range covering concentrations from 0.01 to 2.5 μg/mL to facilitate a more accurate determination of lower concentration. The validation procedures were according to FDA industry guidelines for bioanalytical validation (U.S. Food and Drug Administration, 2018). Blank plasma was used for validation studies.

#### 2.5.1 Linearity

Each aliquot of 50 µL blank plasma with 6 µL formic acid was spiked with pro-EGCG at 0.01, 0.05, 0.1, 0.25, 0.5, 1, 2.5 μg/mL and internal standard pro-EGCG-1–^13^C_8_ at 5.00 μg/mL. The spiked samples were subsequently processed and analyzed by LC–MS as described in Sections 2.3–2.4. The linearity tests were repeated six times. Linearity, R^2^, in each batch was evaluated by the ratio of the peak areas of the pro-EGCG and internal standard against the spiked actual pro-EGCG concentrations in the blank extract.

#### 2.5.2 Reproducibility

Each aliquot of a blank plasma sample was spiked with pro-EGCG at concentrations of 0.01, 0.03, 1.25, 2.5 μg/mL and internal standard at 5 μg/mL to form four levels of QC samples. Each QC sample concentration was calculated by comparing the ratio of areas of pro-EGCG/internal standard peaks to a separate standard curve in the same batch. Intra-day precision at each concentration was calculated by processing six sets of QC samples. Inter-day precision was assessed by calculating three batches of the QC samples of intra-day tests on three different days. Precision was presented as the coefficient of variation (CV).

$$Coefficient\;of\;variation\;\left(CV\right)\;\%=\frac{Standard\;deviation\;ofQC\;samples\;at\;each\;batch}{Mean\;of\;QC\;samples\;at\;each\;batch}\times 100\%$$

#### 2.5.3 Accuracy

Each batch of QC samples contained six sets of plasma samples that were spiked with pro-EGCG at concentrations of 0.01, 0.03, 1.25, and 2.5 μg/mL with internal standard. The concentration of each QC sample was calculated from a separate standard curve. The concentration of each QC sample was compared with the actual spiked concentration. Intra-day accuracy at each level was calculated by processing six sets of the QC samples of the intra-day test. Inter-day accuracy was assessed by three batches of QC samples on three different days.

$$Accuracy\;\left(derivation\;\%\right)=\frac{calculated\;concentration\;of\;QC-actual\;spiked\;concetration}{actual\;spiked\;concentration}\times 100\%$$

#### 2.5.4 Lower limit of quantification of the method

Each aliquot of blank plasma was spiked with 5.00 μg/mL internal standard with decreasing pro-EGCG concentrations. Six samples at each concentration were processed in each batch. Each concentration was evaluated by a separate standard curve in the same batch. The lower limit of quantification (LLOQ) is defined as the lowest concentration of pro-EGCG where reproducibility was < 20% CV whereas the reproducibility of higher concentration QC samples was < 15% CV.

#### 2.5.5 Recovery

Recovery was determined by adopting the method from United Nations Office on Drugs and Crime (UNODC) using internal standard calibration (United Nations Office on Drug and Crime, 2021). Pro-EGCG was spiked into blank samples before extraction and the internal standard was added to the extract after the extraction process. In brief, pro-EGCG was spiked into plasma samples at 0.03, 1.25, and 2.5 μg/mL. After extraction, the internal standard pro-EGCG-1–^13^C_8_ at 5.00 μg/mL was added. The final residue was dissolved in 100 µL methanol and analyzed by LC–MS. The recovered pro-EGCG concentration was calculated by comparing the ratio of the peak areas of pro-EGCG/internal standard to the standard curve as described in the reproducibility. Four batches of samples at each concentration were processed as suggested by the minimum requirement of the UNODC. The recovered rate was equal to recovered concentrations divided by spiked amount × 100%.

$$Recovery\;\left(\%\right)=\frac{calculated\;QC\;concentration\;\left(IS\;spiked\;after\;extraction\right)}{actual\;spiked\;QC\;concentration}\times 100\%$$

#### 2.5.6 Selectivity

Pro-EGCG and internal standard at 1.0 μg/mL and 5.0 μg/mL respectively were analyzed individually using LC–MS to check whether they interfered with each other. Blank plasma samples from 30 SD rats were analyzed to detect any interference at the same m/z and retention time.

#### 2.5.7 Carryover

After 25.0 μg/mL of pro-EGCG solution was injected, two blank plasma extracts were tested to verify any pro-EGCG presented.

#### 2.5.8 Ion suppression/enhancement

The electrospray ionization (ESI) suppression or enhancement effects on pro-EGCG from extracted matrix were tested according to National Association of Testing Authorities (NATA) guidelines and other references (Buhrman et al., 1996; NATA, 2012). Five blank plasma samples from five rats were processed as described above; the final blank plasma extract was spiked with 2.5 μg/mL pro-EGCG and 5.0 μg/mL pro-EGCG internal standard separately. They were injected directly into the LC–MS. The result was obtained by comparing the ion count of pro-EGCG in plasma extract with a pure pro-EGCG solution of the same concentration.

$$Ion\;suppression/enhancement=\frac{ion\;count\;of\;pro-EGCG\;or\;IS\;in\;blank\;extract}{ion\;count\;of\;pro-EGCG\;or\;IS\;in\;methanol}$$

#### 2.5.9 Stability

Three processed plasma samples extracted at 5.0 μg/mL were stored at 4°C for a week. They were tested at t = 0 and 7 days. This duration was selected to ensure the test period is sufficient to cover a batch of samples for analysis in the auto-sampler (Brodie and Hill, 2002; Department of Medical Sciences Ministry of Public Health, 2022). For the long-term storage stability test, six blank plasma samples extract were spiked with pro-EGCG at 5.0 μg/mL and stored at—80°C for a month with three freeze–thaw cycles at the end of each storage. This test period covered common the maximum storage period pending for batches of analysis.

The stability of pro-EGCG in plasma at room temperature was assessed by spiking 50.0 μg/mL pro-EGCG into three sets of 50 µL plasma samples. Each plasma sample was spiked with 5.0 μg/mL pro-EGCG internal standard at time points 0, 3, 5, 10, 20, 30, and 60 min before snap-frozen by liquid nitrogen. The samples were processed as described above and analyzed by UPLC‐Qtof-MS.

We also evaluated the stabilizing effect of different esterase inhibitors. The inhibitors, 4-(2-aminoethyl-)benzenesulponylfluoride HCL (penfabloc), bis-nitophenyl phosphate (BNPP), N-α-tosyl-L-lysine chloromethylketone HCL (TLCK), and dichlorvos, at plasma level of 30 mM; a mixture of sodium fluoride–potassium oxalate (NaF-KO) at plasma level of 10.0 mg/mL and 8.0 mg/mL; and 6 µL formic acid in 50 µL fresh rat plasma sample were used. Subsequently, pro-EGCG was spiked into the blank rat plasma at 5.0 μg/mL in an ice bath for 10 min. An internal standard, 5.0 μg/mL, was added to the plasma samples and snap-frozen by liquid nitrogen at the 10th minute. Three sets of samples were tested. The samples were processed as above and analyzed by UPLC‐Qtof-MS.

The stability of pro-EGCG in plasma with formic acid was assessed by mixing 6 µL of formic acid with 5.0 μg/mL pro-EGCG in 50 µL plasma and stored in an ice bath. Each three plasma samples were spiked with 5.0 μg/mL pro-EGCG internal standard and snap-frozen by liquid nitrogen at time points 0, 10, 15, 20, 30, 45, and 60 min. The samples were analyzed as described.

$$Stability\;\left(\%\right)=\frac{calculated\;concentration\;of\;pro-EGCG\;under\;different\;treatment\;}{actual\;spiked\;concentration}\times 100\%$$

### 2.6 Pharmacokinetic study

Eight groups of three SD rats, about 250 g, were fasted overnight. Pro-EGCG was dissolved in N-methyl-2-pyrrolidone (NMP). A single bolus dose of 50 mg/kg pro-EGCG was given by intragastric administration to each rat. Blood samples from the three rats were collected from the jugular vein at each time point 0, 0.25, 0.5, 1, 2, 6, 8, and 24 h after pro-EGCG administration. Blood samples were temporarily stored at about -10°C in an ice bath within 10 min. The blood samples were centrifuged at -10°C and 1780 × g for 10 min. Each 50 μL of plasma sample was mixed with 6 μL formic acid and subsequently snap-frozen in liquid nitrogen, and stored at –80°C before analysis.

### 2.7 Data analysis

Winonlin 5.1 were used in this study and pharmacokinetic study.

## 3 Results

### 3.1 Validation

Linearity, R^2^, of six calibration curves from 0.01 to 2.5 μg/mL was > 0.999. No non-zero calibrator exceeded ± 15% of the nominal concentration in every curve as required according to the FDA guideline (U.S. Food and Drug Administration, 2018). The coefficient of variation (CV%) of intra-day imprecision was from 1.6 to 8.0% (*n* = 6). The inter-day imprecision of three batches of samples was from 4.03 to 11.56% for the four levels of QC samples: LLOQ, Low (L), Middle (M), High (H), at a concentration of 0.01, 0.03, 1.25, 2.5 μg/mL respectively (Table 1). The imprecision was lower than the FDA requirement for the imprecision of LLOQ to be not greater than 20%, while other concentrations were not greater than 15%. The lower limit of quantification of the method (LLOQ) was 0.01 μg/mL. The deviation of intra-day accuracy (*n* = 6) was from -0.009 to -16.0%, and the deviation of inter-day accuracy of three batches of samples was from 1.77 to -7.92% for the four levels of QC samples (Table 1). The performance well met the FDA limits in that the imprecision should not be greater than 20% for LLOQ and not greater than 15% for the other concentrations. The mean recovery of the method of four batches samples was 50.45 ± 14.36, 94.93 ± 2.86, and 96.63 ± 1.09% (mean + SD) for 0.03, 1.25, 2.5 μg/mL QC samples (Table 2).

**TABLE 1:** Validation results of precision and accuracy. The coefficient of variation was within 20% for the LLOQ and within 15% for the higher concentrations samples in each batch. The deviation from the spiked concentration was within 20% for the LLOQ and within 15% for the higher concentrations. The LLOQ was 0.01 μg/mL.

		Precision (CV %)			Accuracy (deviation %)				
		Intra-batch (*n* = 6)	Inter-batch (*n* = 18)	Intra-batch (Mean ± SD, *n* = 6)	Inter-batch (Mean ± SD, *n* = 18)				
Sample	Conc (µg/mL)	Batch 1	Batch 2	Batch 3	Batch 1–3	Batch 1	Batch 2	Batch 3	Batch 1–3
LLOQ	0.01	1.60	7.99	4.24	11.56	5.17 ± 1.68	−12.98 ± 6.95	−15.94 ± 3.57	−7.92 ± 10.64
Low	0.03	4.16	6.66	7.99	7.21	−0.01 ± 4.16	−8.12 ± 6.12	−7.61 ± 7.38	−5.25 ± 6.83
Middle	1.25	6.90	3.50	4.87	5.13	2.37 ± 7.06	0.99 ± 3.53	3.25 ± 5.025	2.20 ± 5.24
High	2.50	5.61	3.22	3.14	4.03	0.79 ± 5.65	1.53 ± 3.27	2.99 ± 3.23	1.77 ± 4.11

**TABLE 2:** Recovery of four batches of samples at 0.03, 1.25, and 2.5 μg/mL. They were Low, Middle, and High level, respectively.

Sample	Batch 1 (%)	Batch 2 (%)	Batch 3 (%)	Batch 4 (%)	Mean (%) ± SD
Low	42.23	53.64	69.22	36.71	50.45 ± 14.36
Middle	96.70	95.58	96.73	90.72	94.93 ± 2.86
High	96.62	96.97	95.16	97.76	96.63 ± 1.09

**FIGURE 2:**
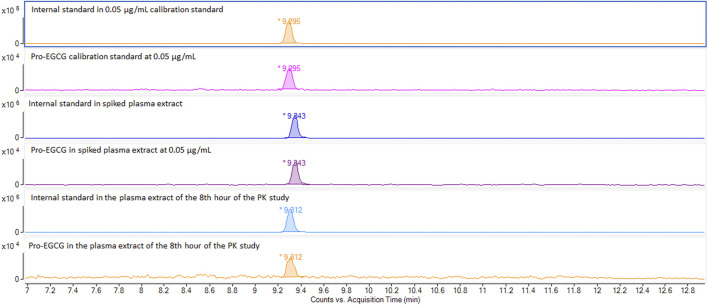
Comparison of exact mass chromatograms of pro-EGCG in calibration standard, spiked plasma extract, and *in vivo* samples. The exact mass chromatogram showed no interferences were detected for the calibration standard at 0.5 μg/mL, spiked plasma samples at 0.5 μg/mL, and *in vivo* plasma sample 8 h after oral administration of pro-EGCG at 50 mg/kg.

As shown in Supplementary Figure S1, the masses 812.2034 m/z and 803.2034 m/z, corresponding to pro-EGCG and pro-EGCG-1–^13^C_8_ internal standard respectively. The sensitivity of pro-EGCG had been increased by 25 folds using ammonium adduct for quantification. Chromatograms of 1.00 μg/mL pro-EGCG and 5.00 μg/mL internal standard pro-EGCG-1–^13^C_8_ showed no interference caused by either compound. The matrix did not incur any interference to the peaks (Supplementary Figure S2). No pro-EGCG and internal standard pro-EGCG-1–^13^C_8_ peaks appeared in blank plasma samples from 30 SD rats. Pro-EGCG could be effectively separated from the plasma matrix. The selected mass could be separated from the matrix masses by the exact mass method using the Q-Tof-MS (Supplementary Figure S3). The exact mass chromatograms in the calibration standard, *in vitro*, and *in vivo* samples are comparable and show no interference (Figure 2).

No carry-over was found in blank plasma after a high level of pro-EGCG, 25.0 μg/mL, was injected. Slight ion enhancement of the extracted matrix was found from 123.9 ± 34.4% and 124.5 ± 29.0% for the five samples of 2.5 μg/mL pro-EGCG and 5.0 μg/mL pro-EGCG-1–13C8 internal standard (Table 3).

**TABLE 3:** Ionization enhancement of pro-EGCG and internal standard in plasma matrix. The table showed the ion counts of 2.5 μg/mL pro-EGCG and 5 μg/mL internal standard separately presented in the standard solutions and plasma matrices.

	Ion counts in standard solution		Ion counts in plasma extract		Ionization enhancements in plasma (%)	
Sample	Pro-EGCG	IS	Pro-EGCG	IS	Pro-EGCG Pl/std	IS Pl/std
S1	10111011	4427436	10704352	4483060	105.9	101.3
S2	9560570	2124117	12001618	2589284	125.5	121.9
S3	14142806	4744239	16850109	5923658	119.1	124.9
S4	5007889	1092030	9018260	1883220	180.1	172.5
S5	19085002	2489394	16980966	2536773	89.0	101.9
Mean					123.9	124.5
SD					34.4	29.0

**FIGURE 3:**
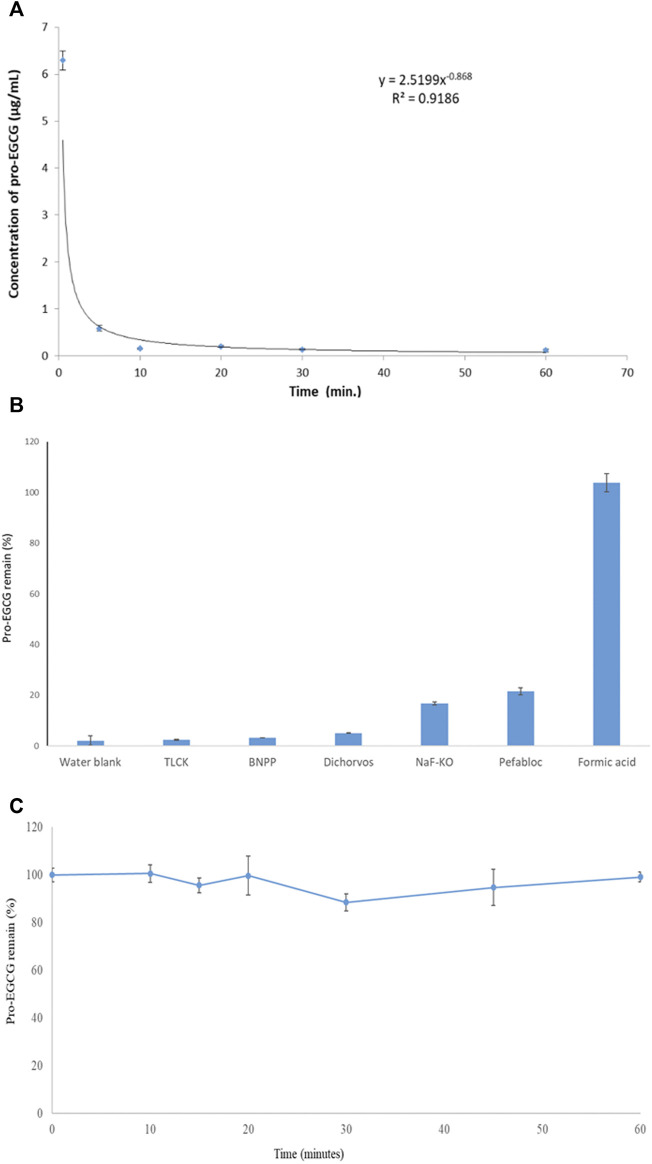
Stability of pro-EGCG in plasma. Diagrams showed **(A)** 50 μg/mL of pro-EGCG was rapidly degraded *in vitro* in plasma (*n* = 6) at room temperature. **(B)** the percentages of efficiency of inhibition of different esterase inhibitors TLCK, BNPP, dichlorvos, and pefabloc, at 30 mM in a mixture of NaF and KO (10 mg/ml and 8 mg/mL) and formic acid at 107 μL/mL for degradation of pro-EGCG in 10 min in an ice bath. **(C)** 6 µL formic acid mixed with 50 µL plasma (about 107 µL formic acid per mL of the mixture) (*n* = 3) to inhibit pro-EGCG degradation in ice (-10°C) over 60 min. Each point was mean ± SD.

Pro-EGCG remained above 95% when 5.0 μg/mL pro-EGCG of processed samples stored at 4°C for 7 days and 4 weeks at—20°C. Pro-EGCG was unstable in plasma at room temperature (Figure 3A). Formic acid was the best stabilizer to preserve pro-EGCG in the plasma compared to other esterase inhibitors. Formic acid treated plasma had 103 ± 3.6% (Mean ± SD, *n* = 3) of pro-EGCG remaining within 10 min in an ice bath while other esterase inhibitors treated plasma had less than 22% of the pro-EGCG left (Figure 3B). Formic acid was able to keep sample molecules stable over 60 min which is sufficient for sample processing (Figure 3C).

### 3.2 Pharmacokinetics study

**FIGURE 4:**
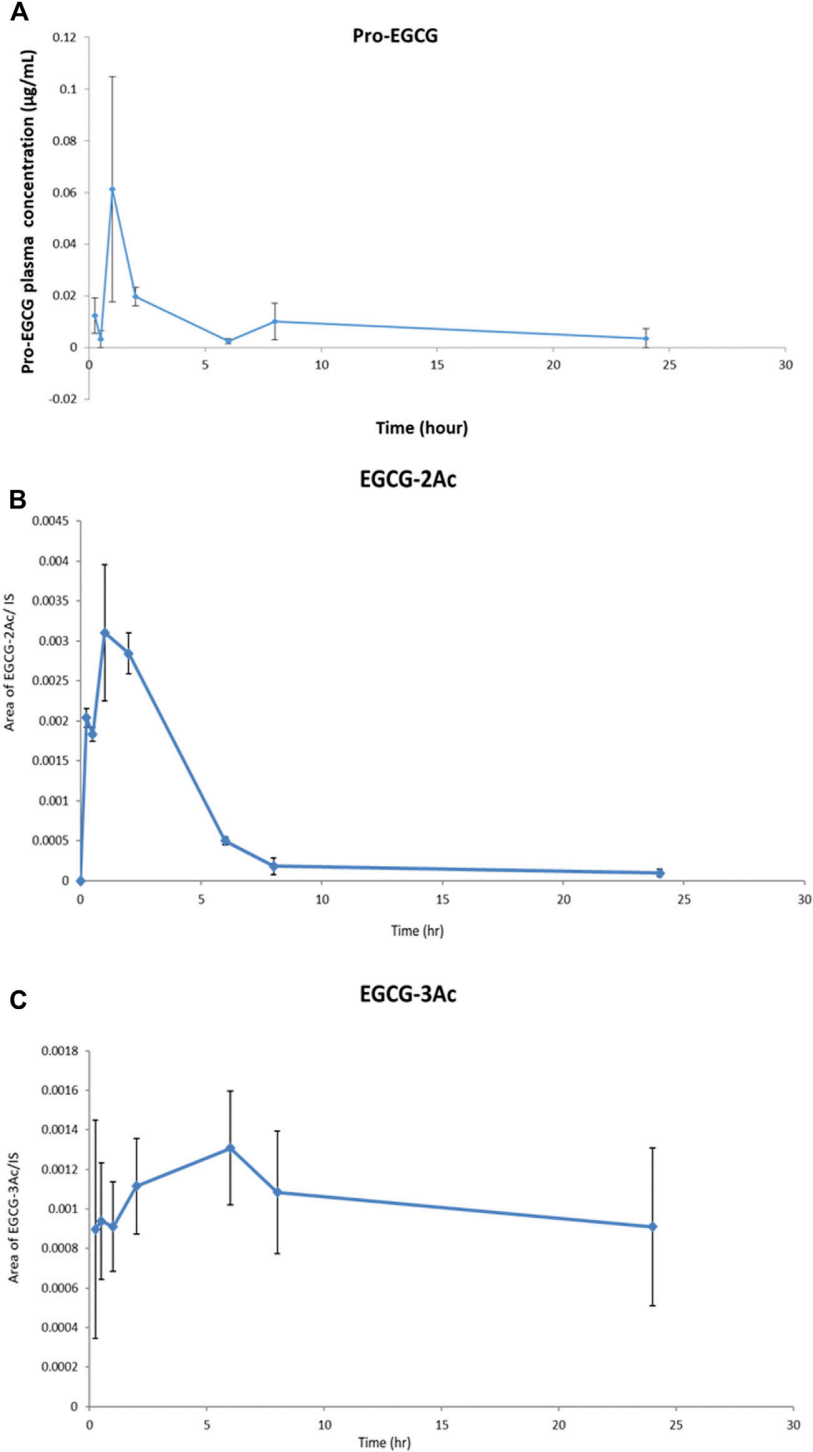
The profiles of Pro-EGCG and its metabolites in plasma. Diagrams showed the levels of **(A)** pro-EGCG, and its metabolites, **(B)** EGCG diacetates (EGCG-2Ac), and **(C)** EGCG triacetates (EGCG-3Ac), (*n* = 3) changed over 24 h after intragastric administration of 50 mg/kg pro-EGCG. Each point was mean ± SE.

Non-compartmental model analysis showed a low level of pro-EGCG, Cmax = 67 ± 0.04 ng/mL and AUC = 0.20 ± 0.05 h × µg/mL, in the rat plasma (*n* = 3) over 24 h of study, and the Tmax was about 1.33 h (Table 4 and Figure 4). The pro-EGCG metabolites, EGCG triacetates (EGCG-3Ac) and EGCG diacetates (EGCG-2Ac), could be detected and quantified. The Tmax of EGCG-2Ac was about 1 h and of EGCG-3Ac about 5 h. However, there are no commercially available standards to confirm these proposed metabolites of EGCG triacetates (EGCG-3Ac) and EGCG diacetates (EGCG-2Ac). In addition, since the ESI + responses of the metabolites were different from the non-polar pro-EGCG, the pharmacokinetic parameters of these metabolites cannot be confirmed.

**TABLE 4:** Pharmacokinetics parameters of pro-EGCG in the plasma of the SD rats (*n* = 3) within 24 h after intragastric administration of 50 mg/kg pro-EGCG.

	Mean ± SE
Tmax (hr)	1.33 ± 0.33
Cmax (µg/mL)	0.067 ± 0.04
Lambda Z (1/hr)	0.20 ± 0.11
AUC (hr*µg/mL)	0.20 ± 0.05
Vz (L/kg)	17282.4 ± 2007.6
Cl_F (mL/hr/kg)	3033575 ± 1515636
MRT (hr)	12.9 ± 6.4

## 4 Discussion

### 4.1 Method improvement

We have modified the extraction and detection methods (Chu et al., 2014) to improve the analytical performance of pro-EGCG measurements. The sample preparation procedures were simplified, the stability of pro-EGCG before extraction was improved, the separation efficiency was increased, the sensitivity of the detection was increased, and its metabolites could be determined.

A narrower linear range was used since the plasma concentration of pro-EGCG was low and a high concentration range for the determination was not necessary. In addition, the narrower range can improve the accuracy when determining a low level of pro-EGCG. Although the concentration of the internal standard was the same as in our previous study, the higher abundance of ammonium adduct of pro-EGCC kept the internal standard in the mid-concentration range to maintain the linearity.

As discussed in the previous publication (Chu et al., 2014), MTBE and isopropanol were used for pro-EGCG extraction and magnesium chloride as an additive. The polar isopropanol was used to penetrate the plasma protein to increase the efficiency of extraction for pro-EGCG. MTBE was used to selectively dissolve the non-polar pro-EGCG and separate the aqueous/organic mixture into two layers. The magnesium chloride solution was used to precipitate the fatty acids out and to keep them in the organic/aqueous interface to minimize the interference by fatty acids. Our previously reported determination for pro-EGCG in plasma was adequate for pharmacokinetic study following intravenous administration (Chu et al., 2014). However, the procedure was laborious because it involved TLC clean-up to remove pro-EGCG from the strongly bound plasma protein. Also, it was not automated. Since pro-EGCG is prone to esterase and gastric fluid hydrolysis, a low plasma level of pro-EGCG following oral administration is expected. Therefore, in this study, we worked out a more sensitive and selective method with minimal degradation during the sample processing procedure. Here, this newly developed method used a higher abundance of the ammonium adduct instead of the hydrogen adduct for pro-EGCG determination to improve the sensitivity. It also used a high-efficiency UPLC core column to separate lipoprotein matrix from pro-EGCG in the plasma extract to replace the labor-intensive TLC clean-up procedure. Pro-EGCG metabolites can also be extracted. The matrix ion enhancement effect was about 24% in this method which is less than the TLC clean-up method at about 40% matrix suppression.

Sensitivity could be further increased by ten times by decreasing the final volume of methanol, increasing the volume of injection to the maximum limit of 10 μL, and increasing the volume of plasma for extraction. However, the matrix effects may also increase. Owing to its hydrophobic nature, pro-EGCG has a strong affinity for plasma proteins. It may be tightly bound to the hydrophobic domain of the protein molecules which may result in low recovery, especially at a low level. Nevertheless, the internal standard helped maintain accuracy and precision. Since FDA guidelines have no specific requirement for recovery in bioanalysis, the recovery rate should be acceptable.

**FIGURE 5:**
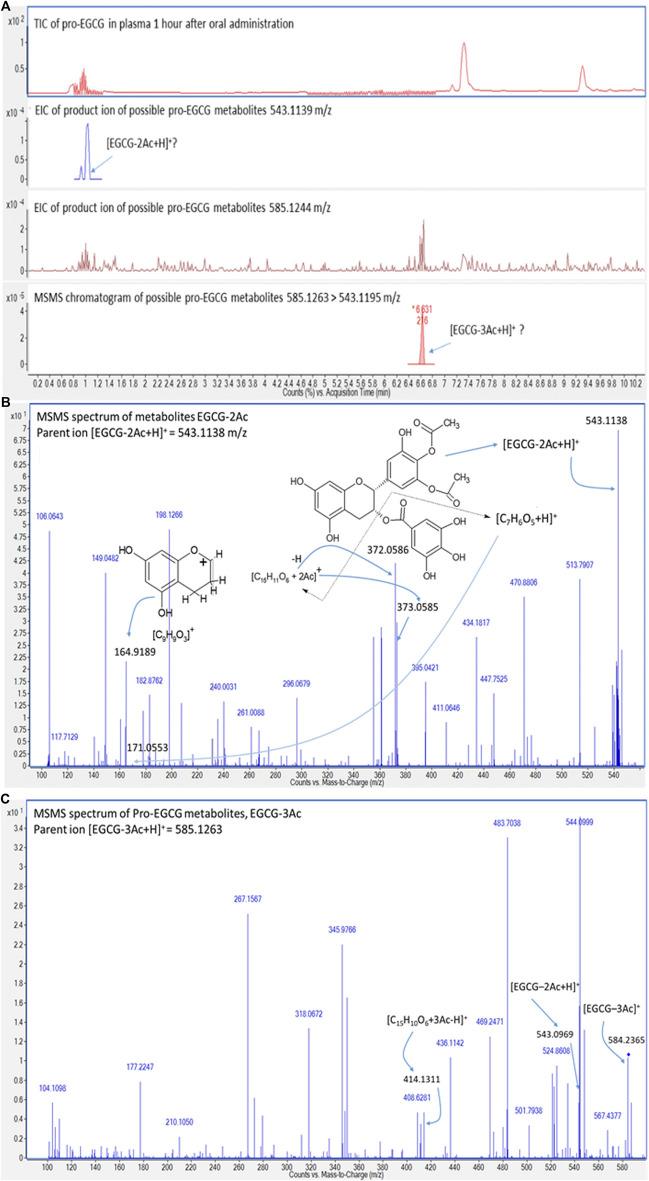
Chromatograms and mass spectra of possible metabolites of pro-EGCG. Diagrams showed **(A)** the chromatograms of TIC and extracted ions of the proposed metabolites, EGCG diacetates [EGCG-2Ac + H]+ (second chromatogram) and EGCG triacetates [EGCG-3Ac + H]+ (third chromatogram) in plasma extract 1 h after oral administration of 50 mg/kg pro-EGCG; **(B)** the MS/MS spectrum of ion 543.1138 m/z supported the peak could be [EGCG-2Ac+H]+; **(C)** possible metabolite, EGCG triacetate, was deduced by the fragmentation of [EGCG--3Ac+H]+, ion mass at 384.2365 m/z, and was further supported by the fourth MS/MS chromatogram.

In addition, since the metabolites of pro-EGCG could be biologically active, like deacetylate analogues of pro-EGCG (Ahmed et al., 2016), it is essential to determine their pharmacokinetics parameters and metabolite profiles to evaluate the efficacy of pro-EGCG. The chromatograms (Figure 5) showed pro-EGCG metabolites, EGCG triacetate and EGCG diacetate, which could be detected by this method. However, since metabolite standards are not commercially available, validation for their determination is not possible.

### 4.2 Validation parameters

Ammonium tends to form adducts with hydrophobic neutral compounds, especially for the species having oxygen atoms (Hua and Jenke, 2012; Sugimura et al., 2017). We selected ammonium as an adduct for pro-EGCG. However, the accuracy data showed the quantified results tended to be lower than the actual amount at a low concentration range although the accuracy still met the FDA requirement. It could be partially explained by a lower recovery rate at low concentration due to the absorption of the matrix. On the other hand, the ammonium adduct is a relatively weak bound adduct having low dissociation energy. The low density of pro-EGCG in the gas phase was less competitive for the gaseous ions than the background matrix in the ESI process. It resulted in a lower abundance of ammonium adducts than the actual amount in a lower concentration range (Westmore and Alauddin, 1986; Yang et al., 2013).

Meanwhile, despite the ammonium adduct having twenty-five times higher intensity than the proton adduct, the limit of detection was only improved 5 times from 0.05 μg/mL to 0.01 μg/mL. Since the limit of detection depended on many factors including extracted matrix, ionization system, the flow rate of elution, type of separating column, and LCMS system, the detection limit cannot be directly compared just by the relative mass intensity.

### 4.3 Pro-epigallocatechin-gallate stability improvement

**FIGURE 6:**
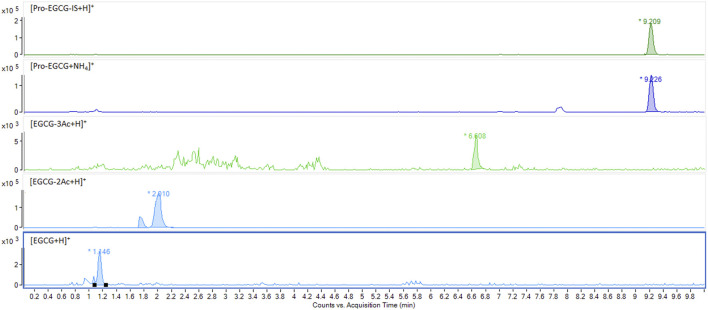
Degradation of pro-EGCG. Chromatograms showed pro-EGCG was degraded forming some typical metabolites like EGCG-3Ac and EGCG-2Ac, and EGCG after 10 min incubation at room temperature.

Although the esters present in the pro-EGCG is stable under an acidic condition like formic acid, it is unstable due to enzymatic degradation in the gastrointestinal tract and blood as demonstrated in Figure 3. Esterase activity causes instability of esterified drugs and their metabolites in biological samples (Satoh et al., 2002; Koitka et al., 2010; Zheng et al., 2012). There are many types of esterases, e.g. carboxylesterase and serum hydrolase, in tissues and blood (Yoshigae et al., 1999). Pro-EGCG is a highly acetylated molecule and susceptible to enzymatic hydrolysis in biological fluids. It is rapidly degraded in plasma at room temperature (Figure 3) into different metabolites (Figure 6). In our sample preparation method, we kept the blood in an ice bath (- 10°C) and rapidly centrifuged it at a low temperature for a short period (– 10°C for 10 min) to minimize the degradation. The plasma sample was then mixed with formic acid which inhibited enzymatic reactions. We have tested different types of non-toxic esterase inhibitors at concentrations higher than other reports (Wei et al., 2013; Cai et al., 2018; Bhuket et al., 2019) by 3–30 folds. We used BNPP and dichlorvos, carboxylesterase inhibitors; TLCK, serine hydrolase inhibitors; Pefabloc, carboxylesterase and serine hydrolase inhibitor; NaF, non-specific inhibitor (Bhuket et al., 2019), but none of them were comparable with formic acid in inhibitory efficiency and reproducibility. Likely the extending structure of acetate groups in pro-EGCG is prone to digestion by many types of esterases that cannot be inhibited. Even a non-specific inhibitor, NaF, cannot effectively inhibit the enzymes. Enzymatic activities can only be inhibited under high acidity due to formic acid, which can maintain stability for at least 60 min as shown in Figure 3C. We have tried to add formic acid to the whole blood during collection to inhibit pro-EGCG degradation. However, the blood coagulated, and the red blood cells lysed immediately so the plasma cannot be obtained.

### 4.4 Pharmacokinetics

Female rats were used in order to match the female age and sex for further pharmacokinetic study of endometriosis treatment. The preliminary pharmacokinetic study of pro-EGCG for SD rats following oral administration showed the bioavailability of pro-EGCG was low as demonstrated in the Cmax, 0.067 ± 0.04 μg/mL, and the AUC, 0.20 ± 0.05 h × µg/mL. It suggests that pro-EGCG has been extensively metabolized. It may be rapidly deacetylated into various acetylated EGCG as supported by the short Tmax, 1.33 h, of pro-EGCG, with rapidly increased levels of proposed metabolites, EGCG-2Ac and EGCG-3Ac, with Tmax at first hour and fifth hour, respectively. On the other hand, the high volume of distribution, Vz, and mean retention time, MRT, indicating that pro-EGCG was possibly extensively distributed at a low level into other compartments.

The *ex vivo* plasma study and *in vivo* pharmacokinetic study showed that pro-EGCG was rapidly and extensively metabolized in the blood. The metabolites appeared to be a better choice for pharmacokinetic study than the parent compound because pro-EGCG needs to undergo extensive *in vivo* metabolism, including phase I and phase 2 conjugation like methylation, sulphation, glucuronidation, and microbial metabolism besides enzymatic de-esterification, before the formation of EGCG. Therefore, we also speculate that EGCG may not be the main product of pro-EGCG *in vivo*. However, we still need the pharmacokinetic data of the parent compound for future clinical studies or trials.

Since the *in vitro* degradation profile is completely different from the *in vivo* metabolism profile due to complicated *in vivo* metabolisms (Cai et al., 2018), we performed *in vivo* pharmacokinetics study directly. Although the pharmacokinetic parameters of the metabolites cannot be determined, their profiles shown in Figure 4 revealed some of their properties. The rate of degradation of pro-EGCG is fast because it contains eight acetyl groups that can be targeted for enzymatic deacetylation. However, EGCG-3Ac is less vulnerable to enzymatic deacetylation. Thus, the rate of digestion of the remaining acetyl group into EGCG-2Ac should be low due to steric hindrance. We presumed the production was mainly due to degradation of EGCG-3Ac which was about 1.25 × 10^−5^ EGCG-3Ac/IS/hr which was lower than the elimination rate of EGCG-2Ac which was about 4.3 × 10–4 EGCG-2Ac/IS/hr. It suggests the production rate of EGCG-2Ac was slow. The level of EGCG-2Ac present in the plasma depends on the rate of production and the rate of elimination of EGCG-2Ac. EGCG-2Ac has higher polarity than EGCG-3Ac (Zhu et al., 2014). Its elimination rate should be higher. If the rate of elimination increases with concentrations, the Tmax of EGCG-2Ac can be earlier than its precursor, EGCG-3Ac. It has been reported that the Tmaxs of curcumin glucuronide and curcumin sulphate were longer than the Tmaxs of their metabolites, dihydrocurcumin glucuronide and dihydrocucumin sulphate due to their higher elimination rates (Fança-Berthon et al., 2021).

Only two metabolites were detected because the bioavailability of pro-EGCG and the metabolites were low, and the matrix affected the detection. Also, we required at least three masses for confident identification. EGCG undetected in the chromatography profile of *in vivo* samples may be because the non-polar extraction mixture, MTBE and IPA, cannot effectively extract the water-soluble compound. In addition, the pro-EGCG and its metabolites should undergo extensive metabolism to EGCG including phase I and phase 2 conjugation like methylation, sulphation, glucuronidation, and microbial metabolism (Cai et al., 2018) besides enzymatic de-esterification. The amount of pure EGCG that can be extracted from the *in vivo* plasma sample can be very small.

The metabolites can be deduced through their molecular masses and fragmentation (Figure 5). The protonated EGCG-2Ac (543.1138 m/z) fragmented into two fragments, a gallate ion, [C7H6O5+H]^+^ ion at 171.0553 m/z and another benzopyran nucleus AC rings linking with the galloyl B ring ion at 373.0585 m/z. The latter ion was further fragmented to benzopyran nucleus ion at 164.9189 m/z, and dehydrogenated to form the [C_15_H_11_O_6_+2Ac]^+^ ion at 372.0585 m/z (Figure 5B). The [EGCG-3Ac]+ ion at 584.2365 m/z, was de-acetylated and protonated to form the [EGCG-2Ac + H]^+^ ion at 543.0969 m/z. The [EGCG-3Ac]+ ion was also protonated and de-gallate to form a benzopyran nucleus AC rings linking with galloyl B ring fragment, [C_15_H_10_O_6_+3Ac-H]^+^ ion at 414.1311 m/z (Figure 5C). Other metabolites cannot be confidently identified and we found that characteristic fragmentation compounds were lacking.

## 5 Conclusion

We have developed a new validated, rapid, robust, sensitive, and reproducible analytical method for the pharmacokinetic study of pro-EGCG following oral administration. The pro-EGCG can be stabilized by the addition of formic acid during plasma sampling and processing by a robotic autosampler capable of large-scale analysis. The pharmacokinetic protocol showed Cmax of 0.067 ± 0.04 μg/mL, Tmax 1.33 h, AUC 0.20 ± 0.05 h × µg/mL, and elimination rate 0.20 ± 0.11 hr^−1^. The pro-EGCG metabolites, EGCG diacetates and EGCG triacetates could be detected with Tmax at 1 h and 5 h respectively. The pharmacokinetic profiles indicated extensive metabolism of the pro-EGCG shortly after oral administration.

## Data Availability

The original contributions presented in the study are included in the article/Supplementary Materials, further inquiries can be directed to the corresponding author.
