# Supplementary material for: Determination of (-)-epigallocatechin-3-gallate octaacetate and its metabolites in plasma of rats for pharmacokinetic study by ultra-performance-liquid-chromatography coupled to quadrupole-time-of-flight-mass-spectrometry
Source: Front Pharmacol. 2022 Oct 11;13:1025053. doi: 10.3389/fphar.2022.1025053 (PMC9592989; doi:10.3389/fphar.2022.1025053)

**Supplementary Figures**

**Figure S1** The chromatograms and mass spectrum of pro-EGCG and internal standard. It showed higher sensitivity of the method by choosing ammonium adduct than proton adduct.


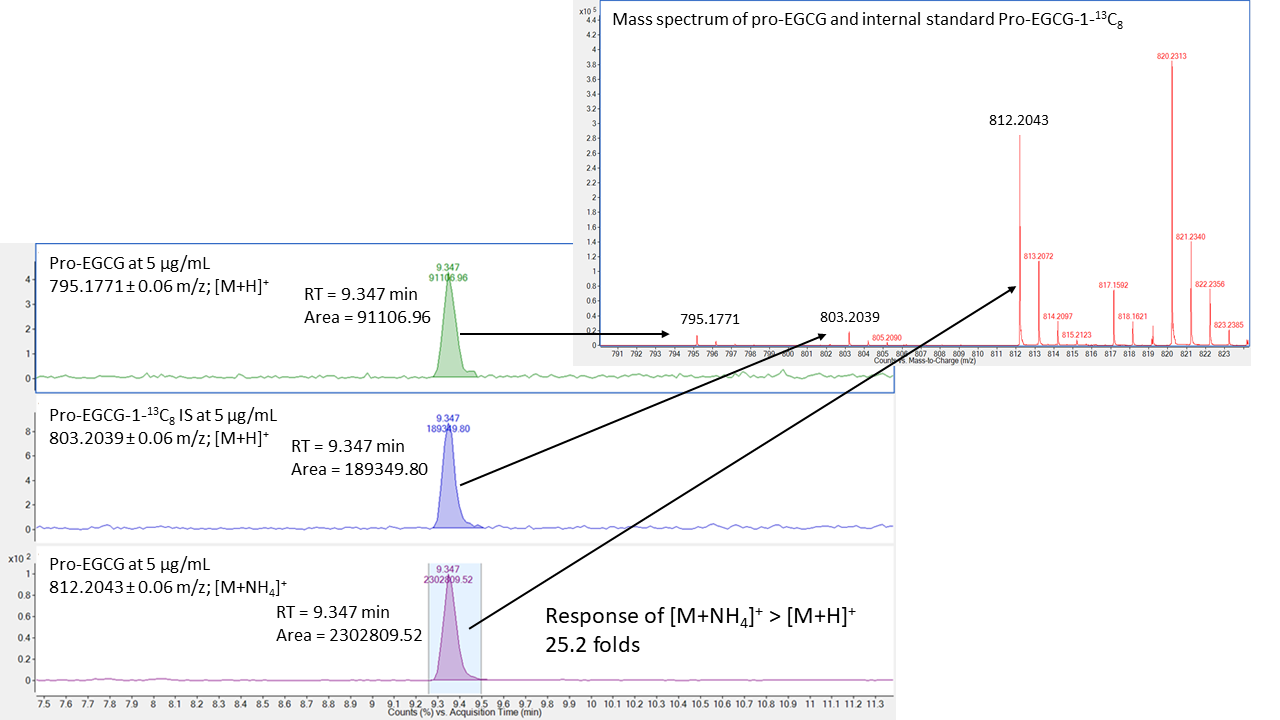


**Figure S2** Chromatograms demonstrated the selectivity of the method. (a) Chromatograms showed only baseline noise in the blank plasma at 803.2039 ± 0.06 m/z and 812.2043 ± 0.06 m/z without interfering peaks at the retention times of pro-EGCG (lower) and pro-EGCG internal standards (upper). (b) Chromatograms showed 1.00 µg/mL standard pro-EGCG and 5.00 µg/mL internal standard pro-EGCG-1-^13^C_8_ individually tested in blank plasma extract. The upper two showed no interference by pro-EGCG on the peak of internal standard, while the lower three showed the internal standard did not produce interfering peaks at the pro-EGCG retention time for the masses of adducts [M+H]^+^ and [M+NH_4_]^+^.

(a)


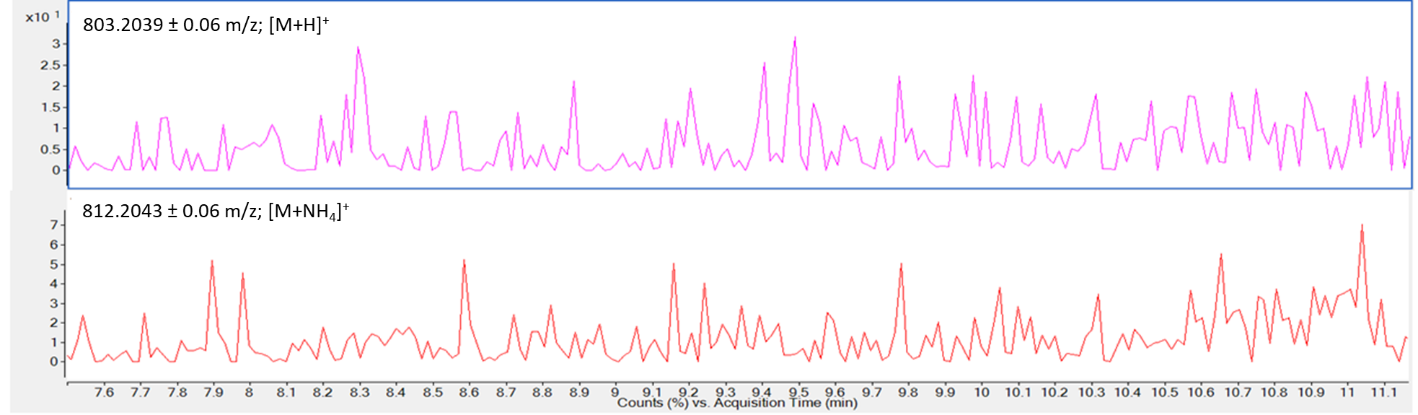


(b)


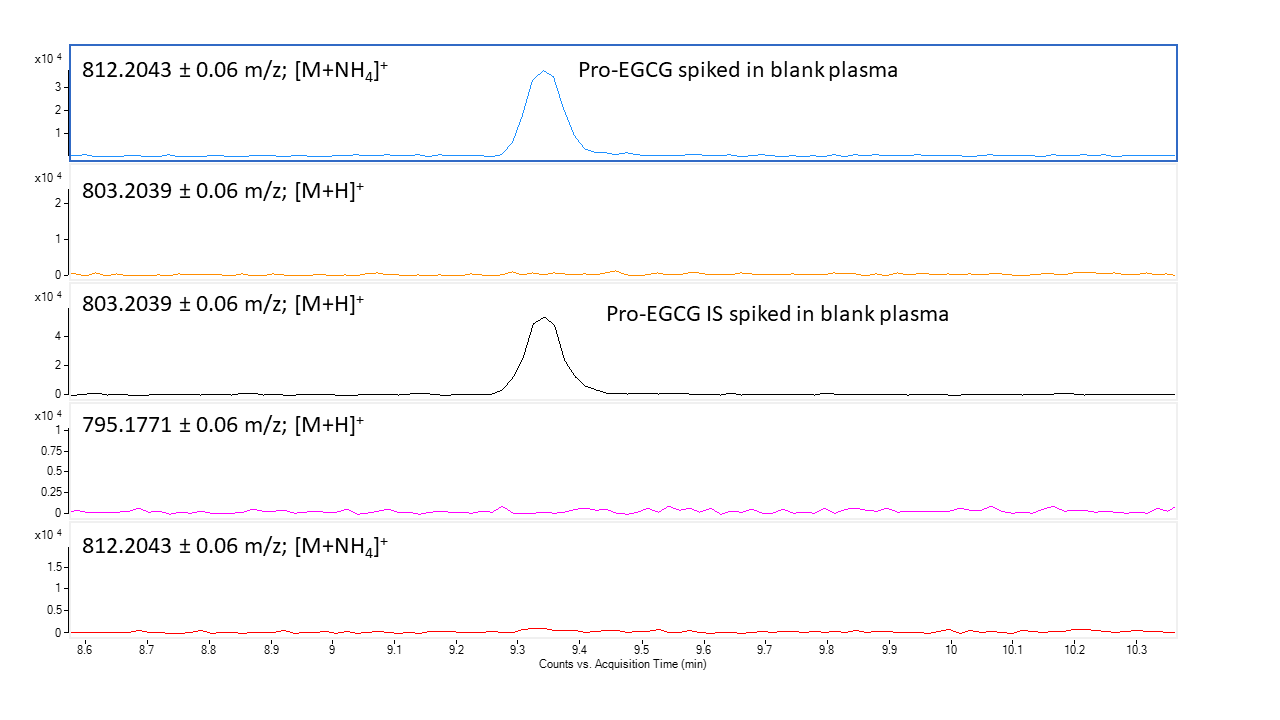


**Figure S3** High selectivity of the method. (a) TIC chromatograms of blank plasma and pro-EGCG spiked plasma at 5 µg/mL showed the ultra-high efficiency of the core UPLC column to separate plasma matrix from pro-EGCG and minimize ion suppression effect due to plasma matrix. (b) The mass spectra of pro-EGCG spiked plasma at 0.01 µg/mL showed the necessity of exact mass quantification to avoid matrix ion interference with an improved signal-to-noise ratio.

(a)


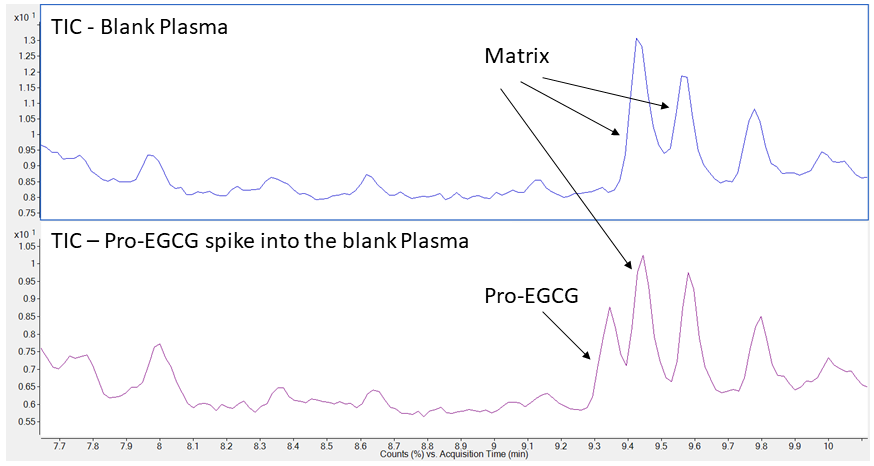


(b)


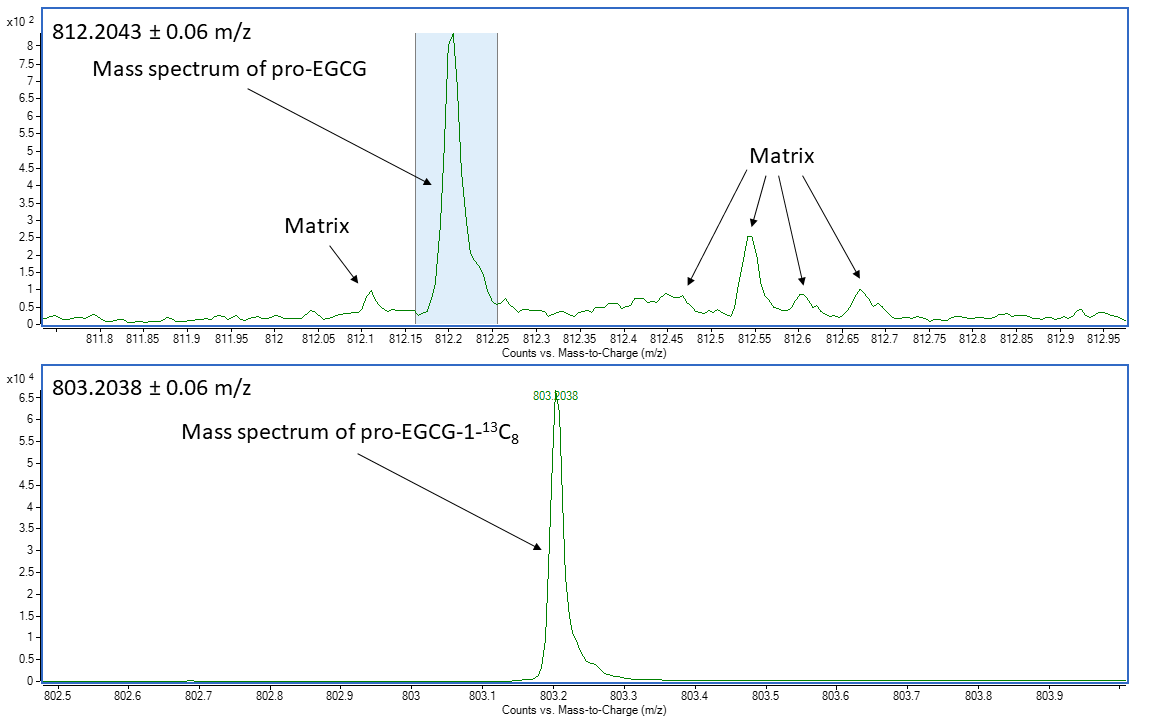

Supplement: Supplementary file 1 [file DataSheet1.docx]
